# *AFF3* upregulation mediates tamoxifen resistance in breast cancers

**DOI:** 10.1186/s13046-018-0928-7

**Published:** 2018-10-16

**Authors:** Yawei Shi, Yang Zhao, Yunjian Zhang, NiJiati AiErken, Nan Shao, Runyi Ye, Ying Lin, Shenming Wang

**Affiliations:** 1 The Department of Breast and Thyroid surgery, the First Affiliated Hospital of Sun Yat-sen University, 58# Zhongshan Two Road, Guangzhou, 510080 Guangdong China; 2 The Department of Vascular surgery, the Third Affiliated Hospital of Sun Yat-sen University, 600# Tianhe Road, Guangzhou, 510000 Guangdong China; 3 The Department of General surgery, the Seventh Affiliated Hospital of Sun Yat-sen University, 628# Zhenyuan Road, Shenzhen, 518100 Guangdong China

**Keywords:** AFF3, Breast cancer, Estrogen receptor-positive, Tamoxifen, Resistance

## Abstract

**Background:**

Although tamoxifen is a highly effective drug for treating estrogen receptor–positive (ER^+^) breast cancer, nearly all patients with metastasis with initially responsive tumors eventually relapse, and die from acquired drug resistance. Unfortunately, few molecular mediators of tamoxifen resistance have been described. Here, we describe *AFF3* (AF4/FMR2 family member 3), which encodes a nuclear protein with transactivation potential that confers tamoxifen resistance and enables estrogen-independent growth.

**Methods:**

We investigated *AFF3* expression in breast cancer cells and in clinical breast cancer specimens with western blot and Real-time PCR. We also examined the effects of *AFF3* knockdown and overexpression on breast cancer cells using luciferase, tetrazolium, colony formation, and anchorage-independent growth assays in vitro and with nude mouse xenografting in vivo.

**Results:**

AFF3 was overexpressed in tamoxifen-resistant tumors. *AFF3* overexpression in breast cancer cells resulted in tamoxifen resistance, whereas RNA interference–mediated gene knockdown reversed this phenotype. Furthermore, *AFF3* upregulation led to estrogen-independent growth in the xenograft assays. Mechanistic investigations revealed that *AFF3* overexpression activated the ER signaling pathway and transcriptionally upregulated a subset of ER-regulated genes. Clinical analysis showed that increased *AFF3* expression in ER^+^ breast tumors was associated with worse overall survival.

**Conclusions:**

These studies establish *AFF3* as a key mediator of estrogen-independent growth and tamoxifen resistance and as a potential novel diagnostic and therapeutic target.

**Electronic supplementary material:**

The online version of this article (10.1186/s13046-018-0928-7) contains supplementary material, which is available to authorized users.

## Background

Breast cancer is one of the three most frequently diagnosed malignancy and the second-leading cause of cancer-related death in women. Every year, an estimated 1 million new cases were diagnosed worldwide [1]. Breast cancer can be divided into subtypes according to histopathological features such as progesterone receptor (PR), estrogen receptor (ER), or erb-b2 receptor tyrosine kinase 2 (ERBB2 or HER2) status, and include ER-positive, HER2-positive, or triple-negative breast cancer [2]. ER-positive (ER^+^) breast cancer constitutes around two-thirds of all breast malignancies [3]. The treatment options for such tumors include targeted anti-hormonal drugs, of which tamoxifen has been the first choice for decades, both in the adjuvant and the recurrent setting [4]. However, approximately 50% of patients with metastatic disease fail to respond, and practically all patients with metastasis with initially responsive tumors eventually develop acquired resistance, which becomes the cause of death [5, 6]. It has been demonstrated that nuclear hormone receptor co-regulator differential expression [7, 8], growth factor signaling crosstalk [9–12], cyclin-dependent kinases (CDKs) [13], CDK inhibitors [14, 15], microRNA regulation [16], and in recent years, acquired *ER* somatic mutations and alterations [17–19], mediate tamoxifen resistance. Although the causes of tamoxifen resistance vary, the most predominant mechanisms are poorly understood. Further insight into the molecular mediators of tamoxifen and hormone therapy resistance would greatly impact the ability to target genes and pathways that could surmount drug resistance, and ameliorate clinical outcomes.

*AFF3* (AF4/FMR2 family member 3, or *LAF4*) was first considered a lymphoid-specific gene; it is expressed and locate in the nuclear of B cells [20]. However, *AFF3* mRNA is nearly imperceptible or in most cases completely absent in plasma cells and many other tissues [20]. *AFF3* encodes a 1227–amino acid protein that is presumed to play a role in transcriptional regulation, as it can directly bind to DNA and contains at least two domains with transactivation activity. These evidence indicated that *AFF3* may be a lymphoid lineage restricted gene with regulated function. *AFF3* shares high sequence identity with the member of AF4/FMR2 family member, such as *AFF1* (AF4/FMR2 family member 1, or *AF4*), *AFF2* (AF4/FMR2 family member 2, or *FMR2*) and *AFF4* (AF4/FMR2 family member 4, or *MCEF*) [21–23]. The *AFF1* gene has been mapped to chromosome 4 and is the target of t(4;11) translocation that occurs in approximately 50% of acute lymphoblastic leukemia (ALL) cases in children aged < 1 year [24, 25], and results in fusion with the *MLL* gene [26]. *MLL* and *AFF4* fusion also occurs in infant ALL but at a much lower frequency. *AFF3* and *MLL* fusion was observed in three independent cases of infant ALL of late [27–29]. Two cases resulted from t(2;11) translocations [27, 28], and the remaining case was due to ins(11,2) insertion [29]. These gene re-arrangement retained the transactivation domain of *AFF3*, and *AFF1* and *AFF4*, suggesting that deregulated expression of the downstream target genes is a common mechanism through which the fusion proteins contribute to oncogenesis. Interestingly, *AFF3* is also deregulated in breast tumors [30]. However, its role in the development of breast cancer and the molecular mechanism underlying its involvement in tumorigenesis remain ambiguous.

In this study, we show that *AFF3* is overexpressed in ER^+^ human breast cancers, leading to tamoxifen resistance and estrogen-independent growth, and that patients with primary breast cancers with *AFF3* overexpression have worse survival. Our study identifies *AFF3* as a new mediator of ER signaling and tamoxifen resistance with potential clinical implications.

## Methods

### Cell culture

Primary normal breast epithelial cell line, MCF-10A, were purchased from the American Type Culture Collection (ATCC, Manassas, VA, USA) and cultured according to the manufacturer’s instruction. Breast cancer cell lines including BT549, HCC1937, MDA-MB231, MDA-MB468, MDA-MB361, T47D, MCF-7 and MDA-MB415, were obtained from the American Type Culture Collection, and grown in DMEM supplemented with 10% fetal bovine serum (FBS), within a humidified atmosphere containing 5% CO_2_ at 37 °C.

### Establishment of resistant cell lines

Parental MCF7 and T47D cells were continuously treated with tamoxifen (Tam, 10^− 7^ M, > 6 months), and the resistant derivatives (TamRes) were selected when the initially sensitive cells resumed comparable growth to the parental cells.

### Tissue specimens

Fresh human tissue samples including 10 breast cancer tissues and 3 normal mammary tissues were collected from the First Affiliated Hospital of Sun Yat-sen University, and were snap frozen and stored at liquid nitrogen until use. A cohort of 101 paraffin-embedded, archived breast cancer specimens was used to determine the clinical significance AFF3, these specimens were clinically diagnosed as breast cancer at from 2000 to 2008. The detail information was shown in Additional file 1: Table S1. For the use of all specimens for research purposes, prior patient’s written informed consents and approval from the First Affiliated Hospital of Sun Yat-sen University.

### RNA extraction and real-time quantitative PCR

Total cellular RNA was extracted using the TRIzol solution (Invitrogen), according to the manufacturer’s protocol. Reverse transcription and Real-time PCR were performed using RT Real-Time™ SYBR Green (Bio-Rad Laboratories, Berkeley, CA, USA). The housekeeping gene *GAPDH* was used as internal controls for mRNAs, respectively. The primers used were as follows: *AFF3* forward: 5′- ACTCAACAGGATGATGGC -3′, *AFF3* reverse: 5′- TGCCTAAAGTGTTCTGGATC -3′; *GAPDH* forward: 5′-GACTCATGACCACAGTCCATGC-3′, *GAPDH* reverse: 3′-AGAGGCAGGGATGATGTTCTG-5′.

### Western blotting

Cellular proteins were prepared in sample buffer [62.5 mM Tris-HCl (pH 6.8), 10% glycerol, 2% SDS] and heated for 10 min at 100 °C. Equal quantities of protein were electrophoresed through a 10% SDS/polyacrylamide gel and transferred to a PVDF membrane (Millipore, Billerica, MA, USA). The membranes were incubated with anti-AFF3 and anti-GAPDH antibodies (1:1,000; Cell Signaling Technology, Danvers, MA, USA), respectively.

### Oligonucleotides, siRNA, plasmids, retroviral infection and transfection

For depletion of AFF3, siRNAs were synthesized and purified by RiboBio (The *AFF3* siRNAs sequence were GGAAGAUGACCUUAAGCUAAG and CAGCUGUGUUGAAGAAAUAAU). Transfection of oligonucleotides and siRNAs were performed using Lipofectamine 3000 (Invitrogen), according to the manufacturer’s protocol. The two human *AFF3*-targeting shRNA sequences were cloned into a pSuper-retro-puro vector to generate pSuper-retro-*AFF3*-RNAi (s). The cDNA of the human *AFF3* gene was amplified by PCR and cloned into a pSin-EF2 lentiviral vector. Stable cell lines expressing AFF3 or AFF3 short hairpin RNAs (shRNAs) were selected by treatment of with 0.5 μg/ml puromycin for 10 days, beginning from 48 h after infection.

### Luciferase assay

Cells were seeded in triplicate in 24-well plates and allowed to settle for 24 h. ER luciferase reporter plasmid pGMER-Lu (YEASEN, SH, CHN) plus 1 ng pRL-TK Renilla plasmid were transfected into the cells using Lipofectamine 3000 Reagent (Invitrogen, Carlsbad, CA, USA). After 48 h transfection, cells were lysed and assayed for luciferase activity using Dual-Luciferase Reporter Assay kit (Promega) according to the manufacturer’s instructions.

### 3-(4, 5-Dimethyl-2-thiazolyl)-2, 5-diphenyl-2H-tetrazolium bromide (MTT) assay

Cells were seeded in 96-well plates. 100 μl of sterile MTT dye (0.5 mg/ml, Invitrogen) was added to the cells at the indicated time points, and the plate was incubated for another 4 h at 37 °C. 150 μl of dimethyl sulfoxide (DMSO) (Sigma) was then added and the absorbance was measured at 570 nm, with 655 nm as the reference wavelength. Three independent experiments were performed and the data were presented as the mean ± SD.

### Colony formation assay

Cells were seeded in a 6-well plate (1 × 10^3^ cells per well) and cultured for 10 days. The colonies were then fixed with 10% formaldehyde for 15 min and stained with 1.0% crystal violet for 5 min. Three independent experiments were performed and the data were presented as the mean ± SD.

### Anchorage-independent growth ability assay

The bottom layer comprising 1% complete medium agar (Sigma) mixture was poured into a well of the 6-well plate. After solidification, cells (1 × 10^3^) were trypsinized and suspended in 2 ml culture medium plus 0.3% agar, and then plated on top of the bottom layer. After 10 days incubation, colony sizes were measured with an ocular micrometer, and colonies greater than 0.1 mm in diameter were counted. The experiment was performed for independently three times for each cell line.

### Mouse xenografts

Xenografts were performed using 5-week-old athymic nude female mice, which were randomly distributed into equal groups (5 mice per group) for each experiment. Mice were injected subcutaneously in the breast with 1 × 10^6^ cells of the indicated cells. Mice were supplemented with estrogen pellets. Tumor volume (mean ± SD; mm^3^) measurements were taken of the palpable tumors every week until week 5 when the mice were sacrificed. The National Institutes of Health Guide for the Care and Use of Laboratory Animals was followed in all experiments.

### Statistical analysis

All data were expressed as the mean ± SD. Student’s *t* test was used to evaluate the significance of the differences between two groups of data in all the pertinent experiments. The *P* value reported was two-sided, and a value of *P* < 0.05 was considered statistically significant.

## Results

### AFF3 upregulation in tamoxifen-resistant (TamR) breast cancers

**Fig. 1:**
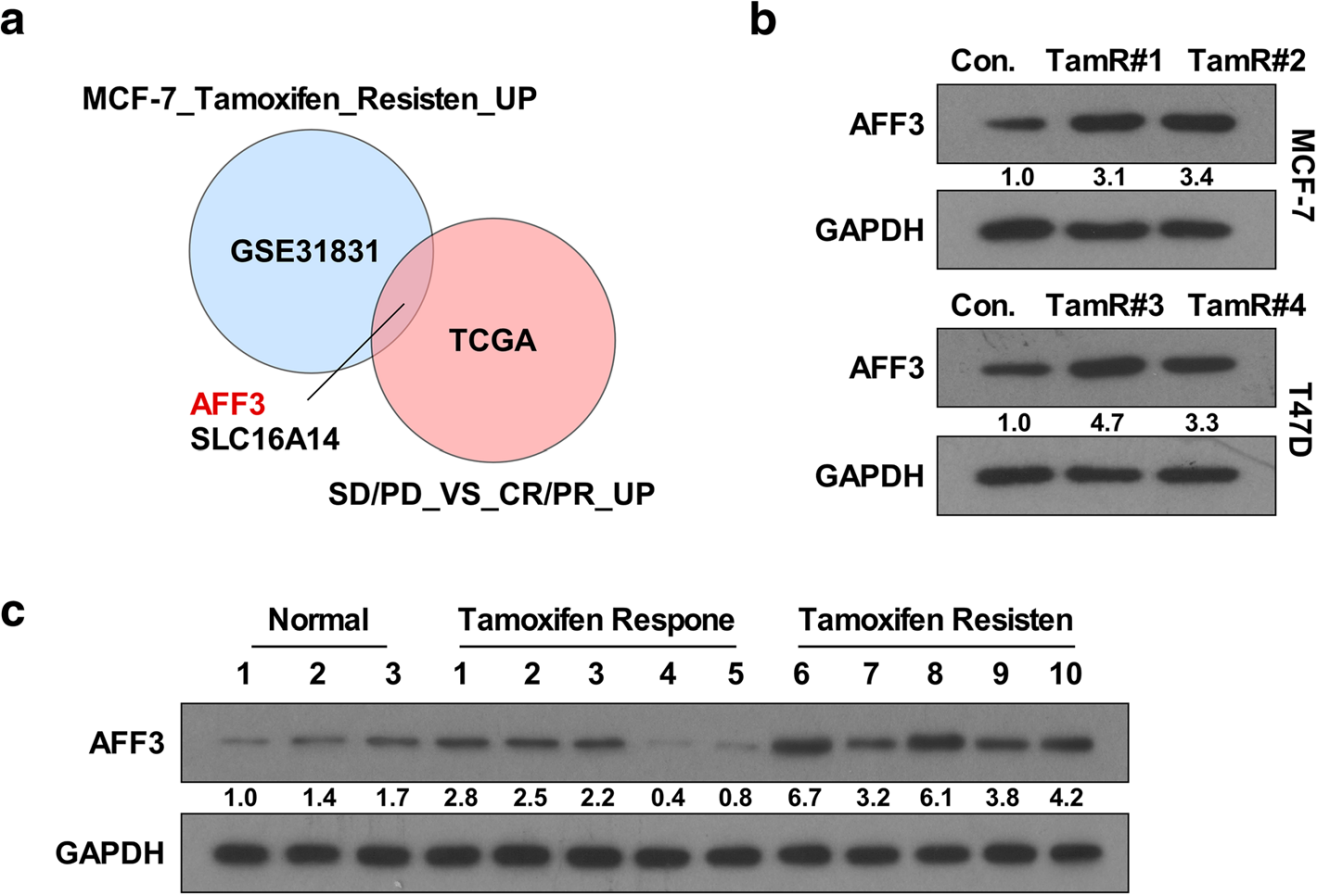
AFF3 upregulation in TamR breast cancers. **a** Venn diagrams showing the overlapping proteins upregulated across two models (*P* < 0.05). **b** Western blotting performed on whole cell lysates demonstrating increased AFF3 protein levels in the TamR cell lines. GAPDH antibody was used as the loading control. **c** Western blotting of AFF3 expression in breast cancer tissues compared with noncancerous breast tissues

*AFF3* and *SLC16A14* (solute carrier family 16 member 14) were upregulated across the subset of genes upregulated in the tamoxifen-resistant (TamR) derivative MCF-7 cell line and the subset of genes overexpressed in patients with incomplete response to chemotherapy (Fig. 1a). Notably, *AFF3* expression was increased in luminal A and luminal B breast cancers, whereas *SLC16A14* expression levels appeared unchanged (Additional file 2: Figure S1a). Quantitative real-time reverse transcription-PCR (qRT-PCR) and western blotting confirmed that AFF3 expression was increased in ER^+^ breast cancer cell lines (Additional file 2: Figure S1b). Subsequently, four TamR cell models were developed independently from the ER^+^ breast cancer cell lines MCF-7 and T47D, and the cell models all exhibited a stable phenotype of sustained cell growth in the presence of estrogen deprivation or tamoxifen (Additional file 2: Figure S1c). Interestingly, AFF3 protein was significantly increased in all four TamR cell models compared with the parental cells in both the MCF-7 and T47D cell lines (Fig. 1b). To verify that AFF3 upregulation was also present in actual human breast cancers, we evaluated specimens from three normal breast tissues and 10 ER^+^ breast cancer tissues. The breast cancer tissues had documented tamoxifen response (patients 1, 2, 3, 4, and 5) and tamoxifen resistance (patients 6, 7, 8, 9, and 10). qRT-PCR and western blotting showed that AFF3 was upregulated in the ER^+^ breast cancer tissues compared to the normal breast tissues. Furthermore, the results confirmed that *AFF3* expression was increased remarkably in the TamR specimens compared to the tamoxifen-responsive samples (Additional file 2: Figure S1d; Fig. 1c). These results demonstrate that *AFF3* overexpression is present in ER^+^ breast cancers and can increase after the development of tamoxifen resistance.

### AFF3 overexpression led to tamoxifen resistance; gene knockdown reversed it

**Fig. 2:**
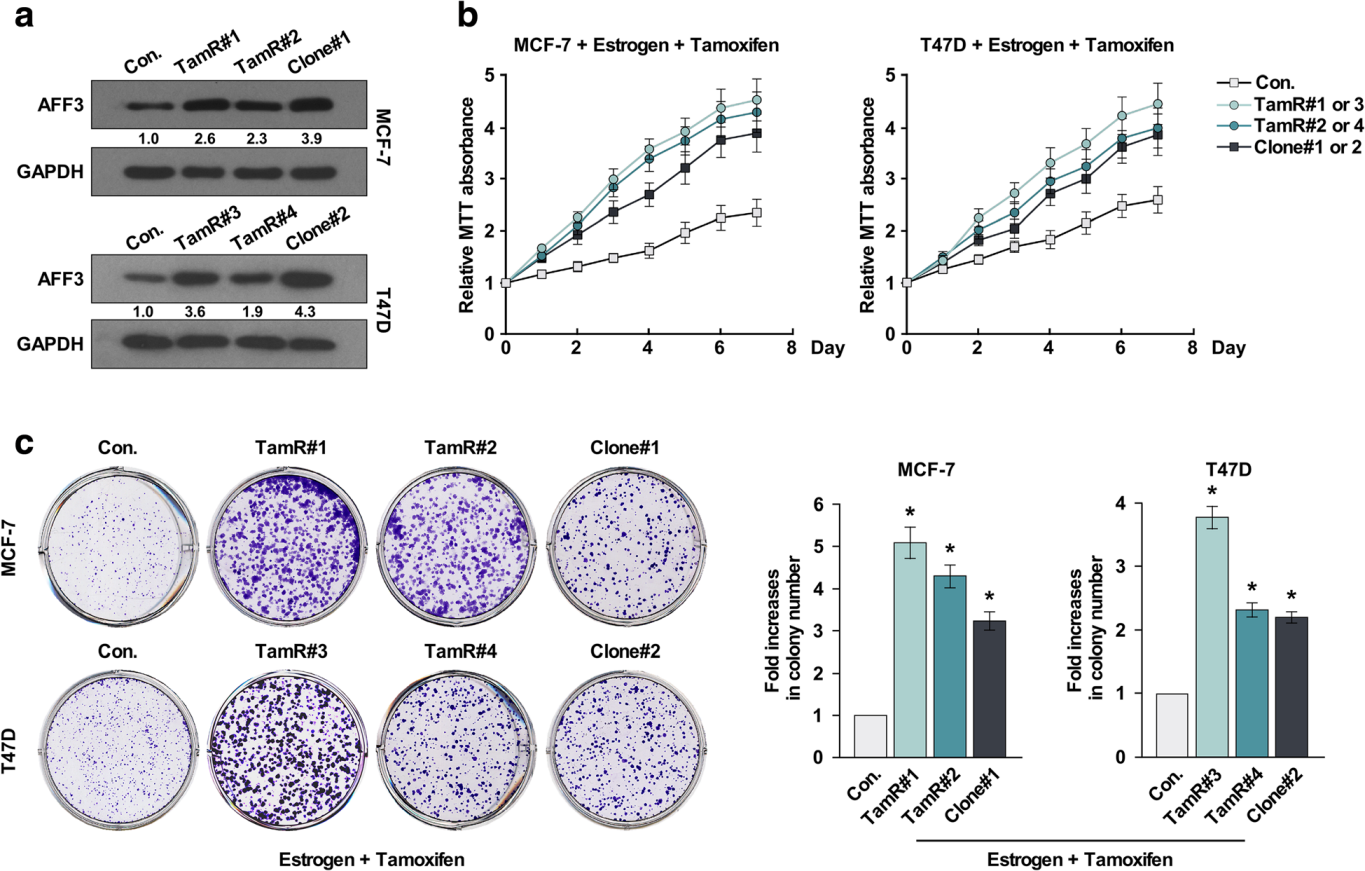
AFF3 overexpression leads to tamoxifen resistance. **a** Whole cell lysates were harvested from cell lines and used for western blotting. GAPDH was used as the loading control. **b** and **c** MTT assay (**b)** and colony formation assay (**c)** of MCF-7 and T47D cells, derived TamR cells and AFF3 overexpressed clones seeded in assay medium and exposed to vehicle (ethanol) and 1 μM 4-OH-tamoxifen for 7 days

To determine whether increased *AFF3* gene expression in the TamR clones would mediate tamoxifen resistance, we overexpressed the full-length *AFF3* complementary DNA (cDNA) in the MCF-7 and T47D cell lines. Western blotting verified AFF3 overexpression in both the TamR and *AFF3*-overexpressing clones compared to the empty vector control cell lines (Fig. 2a). We then exposed the cells to estrogen and tamoxifen for 7 days. Both the TamR and *AFF3*-overexpressing clones demonstrated a TamR phenotype (Fig. 2b and c).

**Fig. 3:**
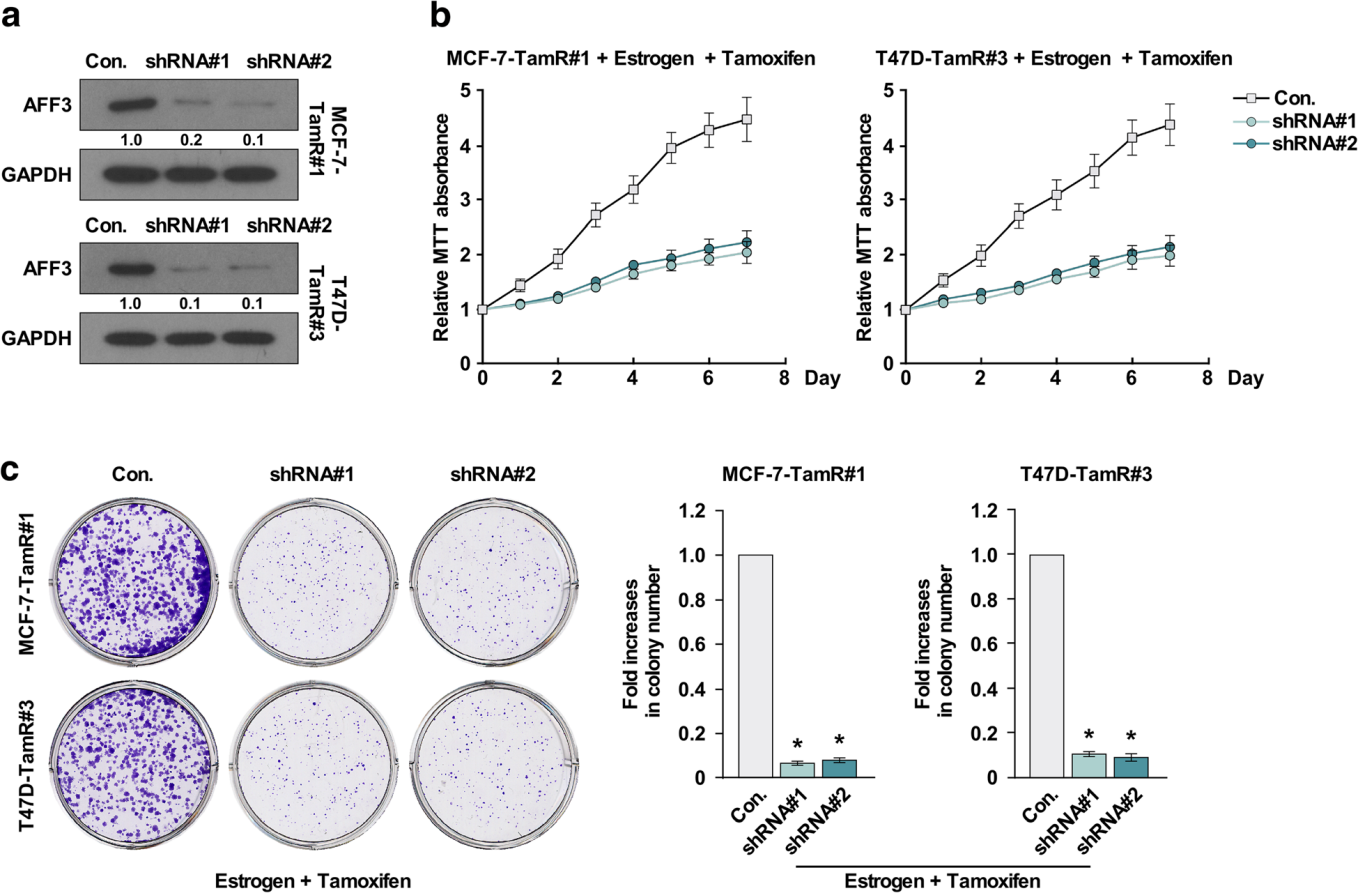
*AFF3* knockdown reverses tamoxifen resistance. **a** Whole cell lysates were harvested from cell lines and used for western blotting. GAPDH was used as the loading control. **b** and **c** MTT assay (**b**) and colony formation assay (**c**) of MCF-7 and T47D derived TamR cells, and their AFF3 knockdown clones seeded in assay medium and exposed to vehicle (ethanol) and 1 μM 4-OH-tamoxifen for 7 days

We next determined whether *AFF3* gene knockdown by RNA interference could reverse tamoxifen resistance. This reversal was accomplished with short hairpin RNA (shRNA) constructs against the *AFF3* transcript in TamR cell lines. We generated two constructs: shRNA#1 and shRNA#2, which were effective in reducing *AFF3* expression when stably expressed in the TamR clones (Fig. 3a). We then assessed tamoxifen sensitivity by growing the *AFF3* knockdown and control cell lines in the presence of estrogen and tamoxifen. The shRNA knockdown reversed the tamoxifen resistance compared with the control TamR clones (Fig. 3b and c). However, *AFF3* knockdown in the parental control cell lines did not improve tamoxifen sensitivity (Additional file 3: Figure S2a). Taken together with the cDNA overexpression experiments, these results strongly suggest that *AFF3* overexpression can mediate a TamR phenotype.

### AFF3 overexpression led to a TamR phenotype in vivo

**Fig. 4:**
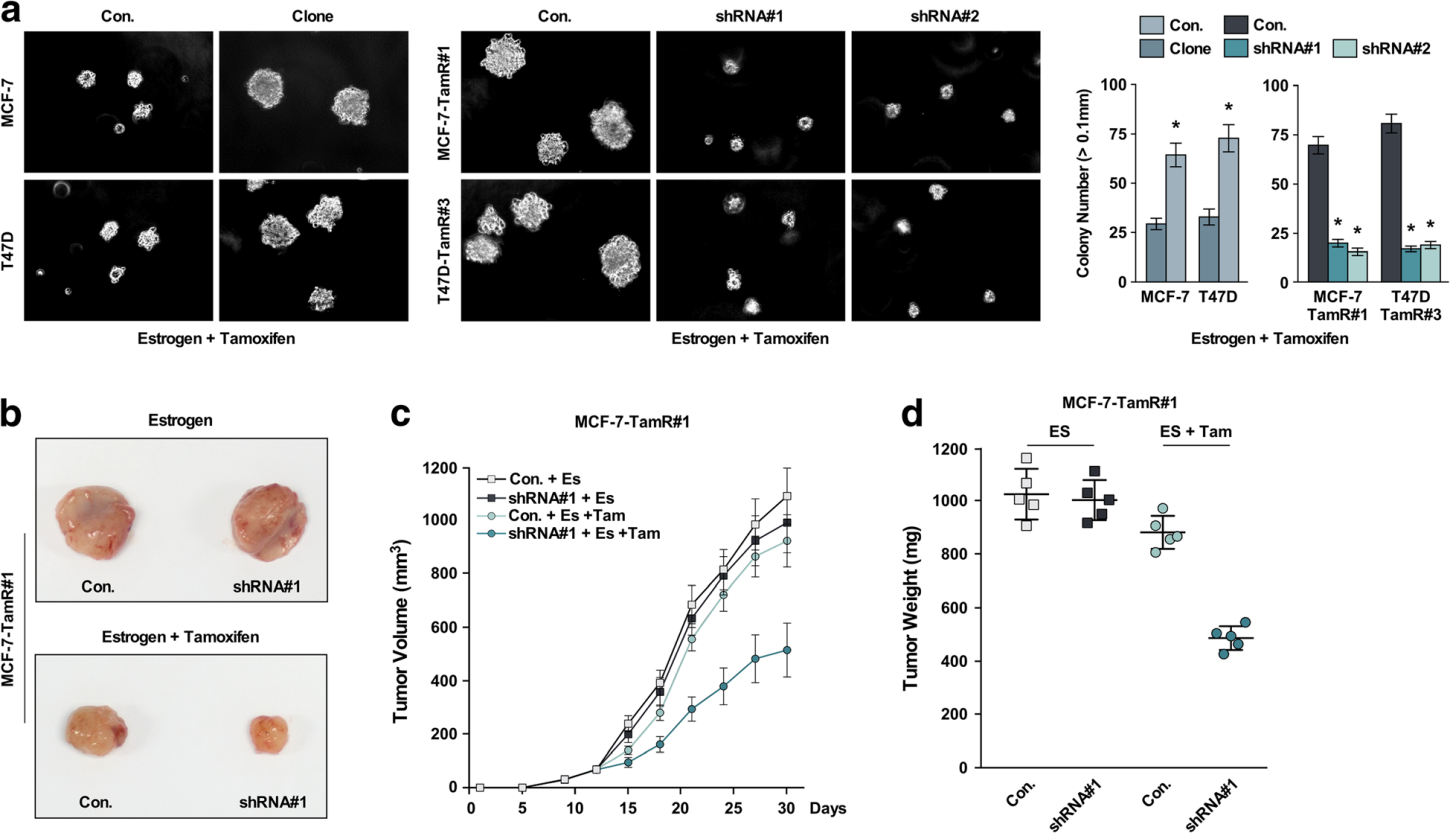
*AFF3* overexpression leads to a TamR phenotype in vivo. **a** Soft agar growth analysis of MCF-7 and T47D cells with *AFF3* overexpression or knockdown (left) and MCF-7 and T47D colony quantification (right). *P < 0.05. **b-d** In vivo tumorigenesis assay of MCF-7-TamR#1 cells with *AFF3* knockdown. **b** Representative images of the tumors in each group; **c** growth curves; **d** tumor weight

We next found that *AFF3* upregulation markedly increased the anchorage-independent growth ability in *AFF3*-overexpressing clones compared with the parental control cell lines. Subsequently, knockdown of *AFF3* expression in the TamR clones decreased their anchorage-independent growth ability (Fig. 4a).

To further characterize *AFF3* mediation of the TamR phenotype, we assessed TamR cell proliferation in xenografts in athymic nude mice. It is well established that MCF-7 cells require estrogen supplementation to grow as xenografts. Control mice inoculated with parental MCF-7 cells did not develop xenograft tumors in the presence of both estrogen and tamoxifen, but the mice inoculated with the *AFF3*-overexpressing clones did (Additional file 2: Figure S2b). Later, we inoculated female athymic nude mice with the TamR clones as well as their *AFF3* knockdown counterparts with or without tamoxifen. There was growth inhibition in vivo following tamoxifen exposure of the TamR clones with *AFF3* shRNA knockdown as compared with the parental TamR clones (Fig. 4b). These results strongly suggest that *AFF3* overexpression can lead to a TamR phenotype.

### AFF3 overexpression activated the ER signaling pathway and increased the expression of ER-regulated genes

**Fig. 5:**
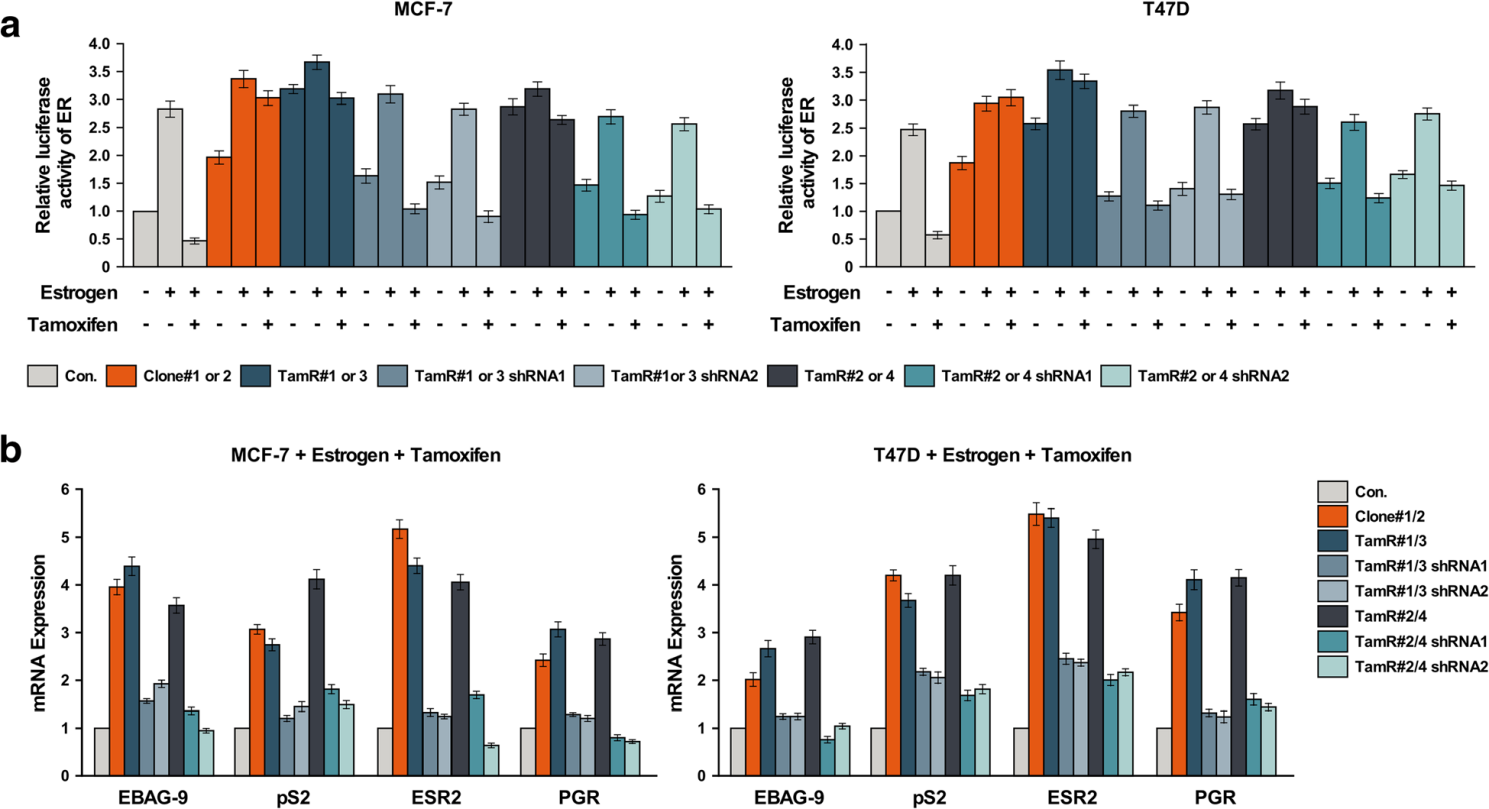
*AFF3* overexpression activates the ER signaling pathway and increases the expression of ER-regulated genes. **a** Luciferase assay indicating the trans-activity of ER in MCF-7 and T47D cells with *AFF3* overexpression or knockdown. *P < 0.05. Bars represent the mean ± SD of three independent experiments. **b** Real-time PCR analysis of mRNA levels of the ER-related genes in MCF-7 and T47D cells with *AFF3* overexpression or knockdown

MCF-7 and T47D cells are frequently used models for ER^+^ estrogen-dependent breast cancer cell growth. However, the TamR clones demonstrated relatively reduced response to exogenous estrogens and indeed were capable of cell proliferation in the absence of estrogen and tamoxifen. We suspected that *AFF3* overexpression may function similarly as an ER coactivator and that its overexpression may lead to increased expression of the ER-regulated genes even in the absence of estrogen. We initially surveyed selected candidate ER-regulated genes using RT-PCR, and demonstrated that, following estrogen inhibition by tamoxifen, gene expression was increased in the *AFF3*-overexpressing clones (Fig. 5a). Conversely, *AFF3* depletion in the TamR clones also reduced the expression of the ER-regulated genes (Fig. 5a). Several growth-promoting genes and enzymes were differentially expressed in the *AFF3* varied clones relative to the control cells, suggesting that *AFF3* diversification may affect ER signaling pathway activity (Fig. 5b).

### Primary luminal breast cancers with AFF3 overexpression had worse prognosis

**Fig. 6:**
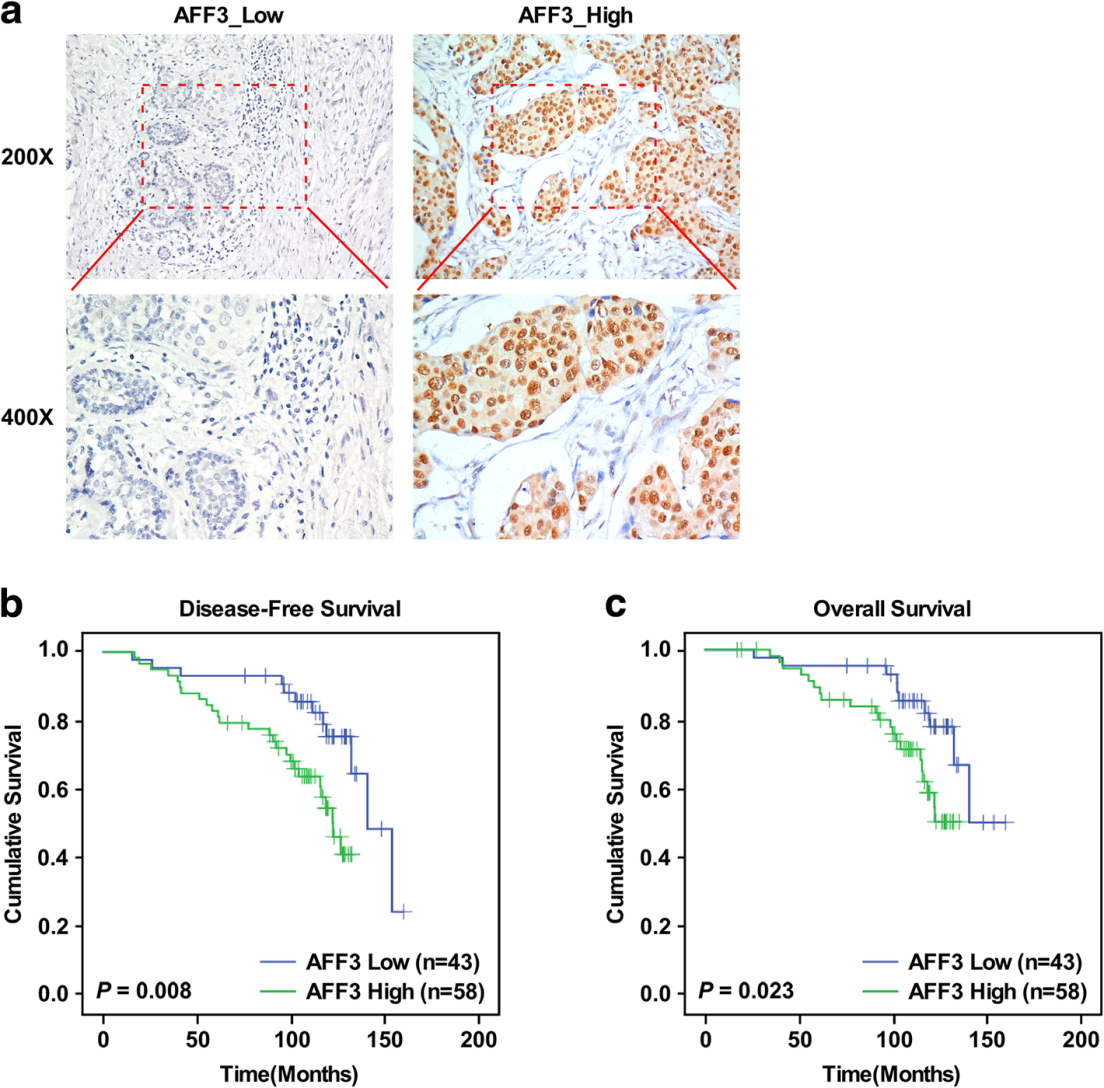
Primary luminal breast cancers with *AFF3* overexpression have worse prognosis. **a** Immunohistochemical staining of AFF3 expression in breast cancer tissues. **b** and **c** Kaplan–Meier survival curves of the relapse-free survival (**b**) and overall survival (**c**) of patients with breast cancer with high or low AFF3 protein levels

To determine the clinical significance of *AFF3* overexpression in human breast cancers, we assessed AFF3 protein levels through immunohistochemical staining on an independent cohort of 101 formalin-fixed, paraffin-embedded tumor tissues from patients with liminal breast cancer (Additional file 1: Table S1). AFF3 positivity (Additional file 1: Table S2) was observed in 95 patients; Cox regression analyses showed a shorter time to progression (TTP) both in univariate (hazard ratio [HR] = 2.634; 95% confidence interval [95% CI]: 2.14–4.16; *P* = 0.010) and multivariate (HR = 2.396; 95% CI: 1.136–5.058; *P* = 0.022) analyses (Additional file 1: Table S3). The presence of AFF3 was significantly associated with shorter survival time in both univariate (HR = 2.449; 95% CI: 2.14–4.16; *P* = 0.027) and multivariate (HR = 2.680; 95% CI: 1.187–6.052; *P* = 0.018) Cox regression analyses (Additional file 1: Table S4). In addition, survival analysis was performed on patients stratified according to *AFF3* levels, with TTP as the endpoint. A significant difference was observed between patients with high AFF3 protein levels (Fig. 6a). AFF3 was positively associated with faster disease progression in luminal breast cancer (*P* = 0.008) (Fig. 6b). An overall survival Kaplan–Meier estimate demonstrated a statistically significant difference between patients whose tumors had high AFF3 compared with those that did not (*P* = 0.023) (Fig. 6c). These results provide additional evidence that *AFF3* overexpression is biologically relevant, has prognostic significance for ER^+^ breast cancers, and may serve as a predictive marker and future target of therapy.

## Discussion

Approximately 75% of breast tumors express ERα, and patients with breast cancer with ERα-positive tumors generally receive endocrine therapy, such as receptor antagonists (antiestrogens), including tamoxifen or fulvestrant, or aromatase inhibitors such as anastrozole or letrozole that inhibit ligand (17β-estradiol) production [31–33]. Notably, tamoxifen has been used to treat both pre- and post-menopausal patients with breast cancer for over 40 years and remains a cornerstone of endocrine therapy for breast cancer. However, intrinsic or acquired resistance to tamoxifen presents a particular clinical concern. Many initially responsive tumors develop resistance to these endocrine therapies, and overall, more women die from ER^+^ breast cancer than from any other molecular subtype [34]. Although there has been much progress in treating ER^+^ breast cancers with endocrine therapies, drug resistance remains a daunting clinical issue. Actually, interest in understanding and uncovering the genetic effectors of endocrine therapy resistance has been renewed with the recent discovery of *ER* mutations and translocations found at relatively high frequency in metastases but that are rare in primary breast tumors. Obviously, these studies suggest that *ER* mutations/alterations emerge after treatment and that there is progression in endocrine therapies. However, they do not throw light on all mechanisms of hormone resistance, and the ability to identify additional mediators of resistance remains of high clinical importance.

The present study demonstrates for the first time that *AFF3* mediates tamoxifen resistance and estrogen-independent growth in breast cancer. We conclude this based on the following: First, independently derived TamR MCF-7 and T47D clones overexpressed *AFF3*. Second, there was *AFF3* upregulation in specimens from patients with tamoxifen-resistant breast cancer. Third, *AFF3* upregulation in two separate ER^+^ breast cancer cell lines led to tamoxifen resistance, and gene knockdown by stable shRNA reversed this phenotype. Fourth, *AFF3* upregulation led to estrogen-independent growth in vivo and upregulated both ER-regulated and non–ER-regulated growth-promoting genes. Finally, patients with ER^+^ (luminal A/B) primary breast tumors with *AFF3* upregulation had significantly worse outcome, in comparison with patients whose primary breast tumors did not upregulate *AFF3*. Our results suggest that the latter may have de novo resistance to tamoxifen and perhaps to other endocrine therapies.

The precise physiological function of AFF3 is not known, but it has been postulated to play a role in lymphoid cell development [20]. The pathological effect of *MLL*-*AFF3* is also not known, but the fact that the transcriptional activation domain of *AFF3*, as well as other members of the *AF4* gene family, is retained in the fusion protein strongly suggests that altered target gene expression is an important step towards ALL pathology. Aberrant expression of the *AFF3* gene itself in mammary epithelial cells could have a similar effect and thereby contribute to the pathogenesis of breast cancer. We demonstrate that *AFF3* is upregulated in breast cancers, leading to tamoxifen resistance and estrogen-independent growth, which are properties more consistent with an oncogene. As a cancer-specific fragile site, it is possible that *AFF3* is lost without any selective pressure for its preservation. However, for ER^+^ breast cancers treated with tamoxifen, the frangibility of this locus allows for overexpression, prompting the emergence of drug-resistant clones. Our results suggest that tamoxifen-insensitive patients have a higher frequency of *AFF3* overexpression, which may mediate estrogen-independent growth. Lack of experimental samples limited our study, as it is not easy to gather metastatic biopsies from patients with clinical follow-up. Regardless, the significantly worse outcomes in patients whose primary breast cancers upregulate *AFF3*, including the large data set from the TCGA (The Cancer Genome Atlas) study, is accord with the idea that *AFF3* may herald intrinsic resistance to tamoxifen therapy with a more aggressive cancer phenotype.

The molecular mediators of endocrine resistance have recently come into much sharper focus, including: i) mutant ER that are constitutively active in the absence of estrogen and more difficult to suppress with conventional endocrine treatment; ii) the upregulation/amplification of ER coactivators that can greatly increase ER activity; iii) the upregulation of alternative oncogenic signaling pathways, including several growth factors and growth factor receptors, resulting in greatly enhanced protein kinase pathway activity; and iv) the amplification or overexpression of gene regions encoding oncogenic proteins and transcription factors, promoting cancer cell survival, invasiveness, and metastasis. Interestingly, the luciferase assays indicated that *AFF3* exerts a profound impact on ER transcriptional activity activation; in a public dataset, we found that *AFF3* expression is related to the ER pathway, suggesting that AFF3 may cause tamoxifen failure by reactivating ER. However, the underlying mechanism by which AFF3 activates ER transcriptional activity and sustains its activity remains unclear, and we are currently investigating it.

## Conclusion

We report *AFF3* gene overexpression in TamR cell models. With the recent interest and success of epigenetic therapies for cancer treatment, it is tempting to speculate that AFF3 may be a druggable protein and that its overexpression may help identify patients whose tumors have intrinsic resistance to tamoxifen and a high-risk phenotype. Therefore, the discovery of a previously unidentified gene, *AFF3*, and its functional role in drug resistance, may lead to improved systemic therapies and predictive markers for treating ER^+^ breast cancers.

## Additional files

Additional file 1: **Table S1.** Clinicopathological characteristics of BC patient samples. **Table S2.** The expression of AFF3 in Breast cancer. **Table S3.** Correlation between AFF3 expression and clinicopathologic characteristics of Breast cancer. **Table S4.** Univariate and multivariate analyses of various prognotic parameters in patients with BC Cox-regression analysis. **Table S5.** Univariate and multivariate analyses of various prognotic parameters in patients with BC Cox-regression analysis. (DOCX 27 kb)

Additional file 2: **Figure**. **S1.** **a** Expression level of AFF3 and SLC116A14 in breast cancer tissues compared with noncancerous breast tissues (*n* = 627; TCGA). **b** Real-time PCR analysis and western blot analysis of AFF3 expression in normal breast cell MCF10A and breast cancer cell lines, including BT-549, HCC1937, MDA-MB231, MDA-MB468, MDA-MB361, T47D, MCF-7, MDA-MB-415. **c** MTT assay of MCF-7 and T47D cells, derived TamR cells seeded in assay medium and exposed to vehicle (ethanol) or 1 μM 4-OH-tamoxifen for 7 days. **d** Real-time PCR analysis of AFF3 expression in breast cancer tissues compared with noncancerous breast tissues. (TIF 1817 kb)

Additional file 3: **Figure**. **S2.** **a** Colony formation assay of MCF-7 and T47D cells, derived AFF3 knock down clones seeded in assay medium and exposed to vehicle (ethanol) and 1 μM 4-OH-tamoxifen for 7 days. **b** Representative images of the tumors in each group, growth curves and tumor weight. (TIF 8999 kb)

## Abbreviations

AFF1 or AF4: AF4/FMR2 family member 1
AFF2 or FMR2: AF4/FMR2 family member 2
AFF3: AF4/FMR2 family member 3
AFF4 or MCEF: AF4/FMR2 family member 4
ALL: acute lymphoblastic leukemia
ATCC: the American Type Culture Collection
CDKs: cyclin-dependent kinases
DMSO: dimethyl sulfoxide
ER: estrogen receptor
ER^+^: estrogen receptor–positive
ERBB2 or HER2: erb-b2 receptor tyrosine kinase 2
MTT: 3-(4, 5-Dimethyl-2-thiazolyl)-2, 5-diphenyl-2H-tetrazolium bromide
PR: progesterone receptor
TamR: tamoxifen-resistant
