# Supplementary material for: AFF3 upregulation mediates tamoxifen resistance in breast cancers
Source: J Exp Clin Cancer Res. 2018 Oct 16;37:254. doi: 10.1186/s13046-018-0928-7 (PMC6192118; doi:10.1186/s13046-018-0928-7)
Supplement: Supplementary file 1 — Table S1. Clinicopathological characteristics of BC patient samples. Table S2. The expression of AFF3 in Breast cancer. Table S3. Correlation between AFF3 expression and clinicopathologic characteristics of Breast cancer. Table S4. Univariate and multivariate analyses of various prognotic parameters in patients with BC Cox-regression analysis. Table S5. Univariate and multivariate analyses of various prognotic parameters in patients with BC Cox-regression analysis. (DOCX 27 kb) [file 13046_2018_928_MOESM1_ESM.docx]

**Supplementary Information**

**Supplementary Methods**

**RNA extraction, quantitative real-time PCR (qRT -PCR)**

*EBAG-9* forward primer: 5’-AGCTGCTCTGGGTACTGTTT-3’; *EBAG-9* reverse primer:5’- GGCAGTAAGGTCACGCAAAT -3’; *pS2* forward primer: 5’- GGAACTGGGTCGTGGAAGGAT-3’; *pS2* reverse primer: 5’- AGAAGAACAAAACGCCCCCG-3’; *ESR2* forward primer: 5’- CCGATGCTTTGGTTTGGGTG -3’; *ESR2* reverse primer : 5’- GAGCAGATGTTCCATGCCCT-3’; *PGR* forward primer: 5’- GTATTTGTGCGTGTGGGTGG-3’; *PGR* reverse primer: 5’- CCGCCTCGGGTTGTAGATTT-3'. Expression data were normalized to the geometric mean of housekeeping gene *GAPDH* to control the variability in expression levels and calculatedas 2^-[(Ct of gene)-(Ct of^ *^GAPDH^*^)]^, where Ct represents the threshold cycle for each transcript.

**Table S1 Clinicopathological characteristics of BC patient samples**

|  | **Number of cases** |
| --- | --- |
| **Age(years)** |  |
| ≤ 51 | 52 |
| > 51 | 49 |
| **Menopause** |  |
| Yes | 48 |
| No | 53 |
| **Clinical Stage** |  |
| I | 26 |
| IIa | 29 |
| IIb | 30 |
| IIIa | 10 |
| IIIb | 3 |
| IV | 3 |
| **T classification** |  |
| T1 | 37 |
| T2 | 50 |
| T3 | 10 |
| T4 | 4 |
| **N classification** |  |
| N0 | 46 |
| N1 | 38 |
| N2 | 8 |
| N3 | 4 |
| N4 | 5 |
| **M classification** |  |
| Yes | 3 |
| No | 98 |
| **Molecular classification** |  |
| Luminal A | 75 |
| Luminal B | 26 |
| **Survive or Mortality** |  |
| Survive | 70 |
| Mortality | 31 |

**Table S2 The expression of AFF3 in Breast cancer**

| **Expression of AFF3** |  |
| --- | --- |
| Negative | 6(5.9%) |
| Positive | 95 (94.1%) |
| Low expression | 43 (42.6%) |
| High expression | 58 (57.4%) |

**Table S3 Correlation between AFF3 expression and clinicopathologic characteristics of Breast cancer**

| **Characteristics** | | **AFF3** | | **Chi-square test**  ***P*-value** | **Fisher’s Exact**  **test *P*-value** |
| --- | --- | --- | --- | --- | --- |
|  |  | **Low No. cases** | **High No. cases** |  |  |
| **Age (years)** | ≤ 51 | 22 | 30 | 0.955 | 1.000 |
|  | > 51 | 21 | 28 |  |  |
| **Menopause** | Yes | 22 | 26 | 0.528 | 0.552 |
|  | No | 21 | 32 |  |  |
| **Clinical Stage** | I~II | 38 | 47 | 0.318 | 0.413 |
|  | III~IIV | 5 | 11 |  |  |
| **T classification** | T1~T2 | 39 | 48 | 0.254 | 0.383 |
|  | T3~T4 | 4 | 10 |  |  |
| **N classification** | N0 | 23 | 23 | 0.167 | 0.225 |
|  | N1~N4 | 20 | 35 |  |  |
| **M classification** | No | 41 | 57 | 0.392 | 0.573 |
|  | Yes | 2 | 1 |  |  |
| **Molecular classification** | Luminal A | 34 | 41 | 0.341 | 0.368 |
|  | Luminal B | 9 | 17 |  |  |
| **Survive or Mortality** | Survive | 33 | 37 | 0.163 | 0.194 |
|  | Mortality | 10 | 21 |  |  |

**Table S4 Univariate and multivariate analyses of various prognotic parameters in patients with BC Cox-regression analysis**

|  | **Univariate analysis** | | | **Multivariate analysis** | | |
| --- | --- | --- | --- | --- | --- | --- |
|  | **No. patients** | ***P*** | **Regression coefficient (SE)** | ***P*** | **Relative risk** | **95% confidence interval** |
| **Molecular classification** |  | | | | | |
| **Luminal A** | 75 | 0.026 | 2.186(0.352) | 0.021 | 2.322 | 1.138-4.738 |
| **Luminal B** | 26 |  |  |  |  |  |
| **N classification** |  | | | | | |
| **N0** | 46 | 0.001 | 3.606(0.387) | 0.030 | 2.431 | 1.088-5.432 |
| **N1~N4** | 55 |  |  |  |  |  |
| **Clinical Stage** |  | | | | | |
| **I~II** | 85 | 0.001 | 3.846(0.358) | 0.003 | 3.133 | 1.486-6.606 |
| **III~IV** | 16 |  |  |  |  |  |
| **Expression of AFF3** |  | | | | | |
| **Low expression** | 43 | 0.010 | 2.634(0.376) | 0.022 | 2.396 | 1.136-5.058 |
| **High expression** | 58 |  |  |  |  |  |

**Table S5 Univariate and multivariate analyses of various prognotic parameters in patients with BC Cox-regression analysis**

|  | **Univariate analysis** | | | **Multivariate analysis** | | |
| --- | --- | --- | --- | --- | --- | --- |
|  | **No. patients** | ***P*** | **Regression coefficient (SE)** | ***P*** | **Relative risk** | **95% confidence interval** |
| **T classification** |  | | | | | |
| **T1~T2** | 87 | 0.001 | 3.786(0.399) | 0.001 | 4.088 | 1.848-9.041 |
| **T3~T4** | 14 |  |  |  |  |  |
| **M classification** |  | | | | | |
| **Yes** | 3 | 0.049 | 4.287(0.739) | 0.010 | 7.043 | 1.584-31.313 |
| **No** | 98 |  |  |  |  |  |
| **Expression of AFF3** |  | | | | | |
| **Low expression** | 43 | 0.027 | 2.449(0.406) | 0.018 | 2.680 | 1.187-6.052 |
| **High expression** | 58 |  |  |  |  |  |
