# Supplementary figures and images for: AFF3 upregulation mediates tamoxifen resistance in breast cancers
Source: J Exp Clin Cancer Res. 2018 Oct 16;37:254. doi: 10.1186/s13046-018-0928-7 (PMC6192118; doi:10.1186/s13046-018-0928-7)

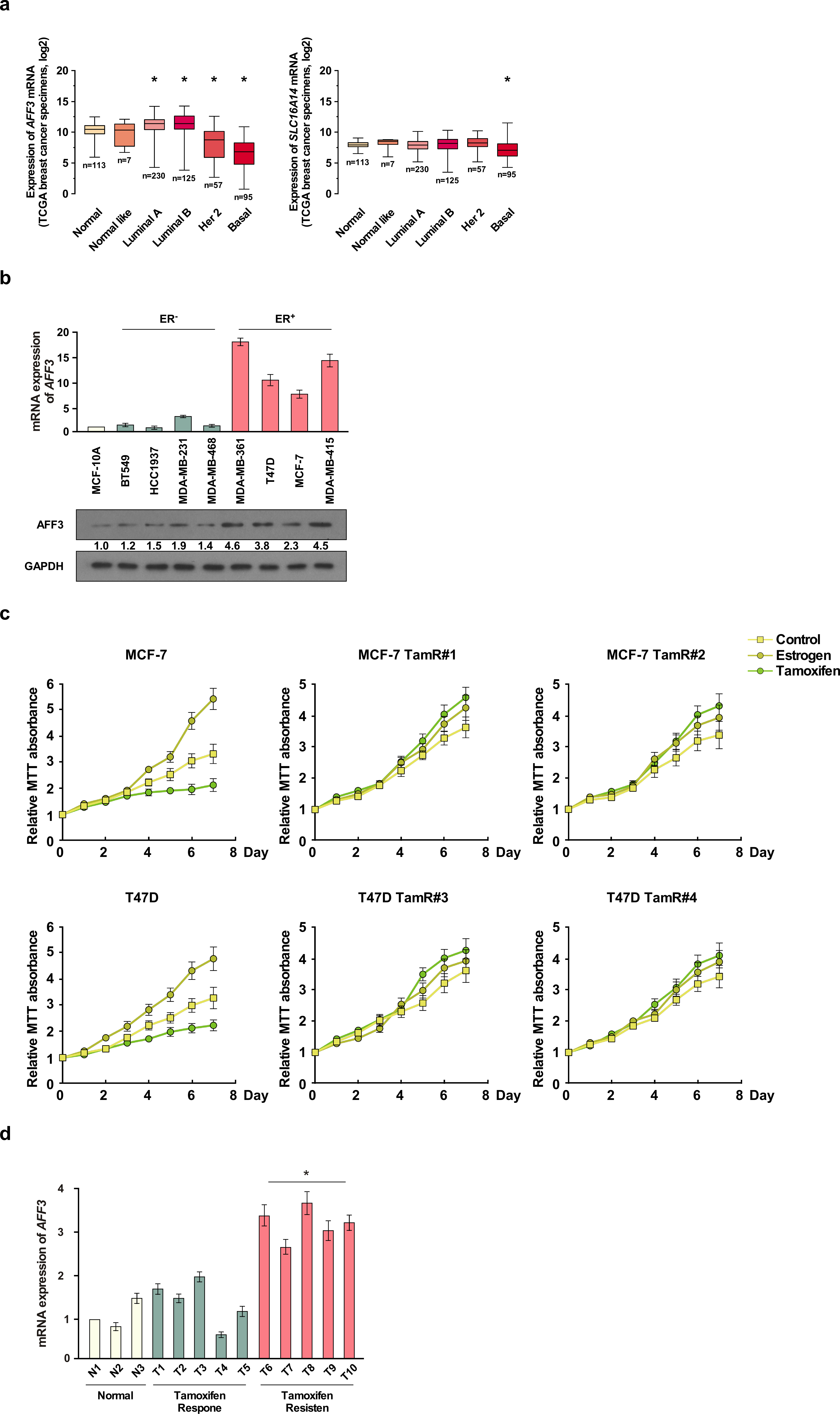

Supplement: Supplementary file 2 — Figure. S1. a Expression level of AFF3 and SLC116A14 in breast cancer tissues compared with noncancerous breast tissues (n = 627; TCGA). b Real-time PCR analysis and western blot analysis of AFF3 expression in normal breast cell MCF10A and breast cancer cell lines, including BT-549, HCC1937, MDA-MB231, MDA-MB468, MDA-MB361, T47D, MCF-7, MDA-MB-415. c MTT assay of MCF-7 and T47D cells, derived TamR cells seeded in assay medium and exposed to vehicle (ethanol) or 1 μM 4-OH-tamoxifen for 7 days. d Real-time PCR analysis of AFF3 expression in breast cancer tissues compared with noncancerous breast tissues. (TIF 1817 kb) [file 13046_2018_928_MOESM2_ESM.tif]

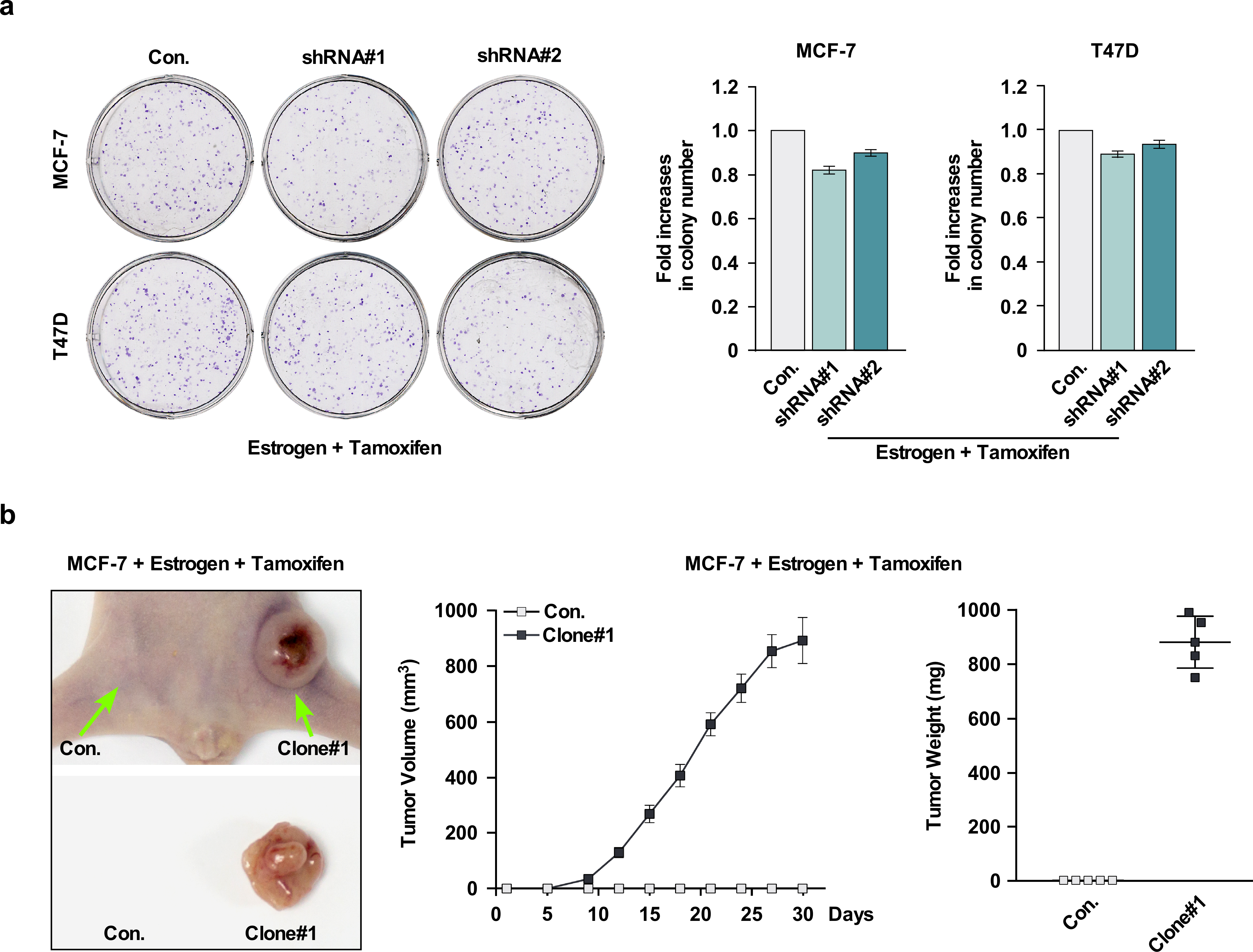

Supplement: Supplementary file 3 — Figure. S2. a Colony formation assay of MCF-7 and T47D cells, derived AFF3 knock down clones seeded in assay medium and exposed to vehicle (ethanol) and 1 μM 4-OH-tamoxifen for 7 days. b Representative images of the tumors in each group, growth curves and tumor weight. (TIF 8999 kb) [file 13046_2018_928_MOESM3_ESM.tif]
